# Intravenous to oral transition of antibiotics for gram-negative bloodstream infection at a University hospital in Thailand: Clinical outcomes and predictors of treatment failure

**DOI:** 10.1371/journal.pone.0273369

**Published:** 2022-09-22

**Authors:** Titawadee Pradubkham, Gompol Suwanpimolkul, Alan Edward Gross, Chotirat Nakaranurack

**Affiliations:** 1 College of Pharmacotherapy Thailand, Nontaburi, Thailand; 2 Faculty of Medicine, Division of Infectious Diseases, Department of Medicine, Chulalongkorn University, King Chulalongkorn Memorial Hospital, Thai Red Cross Society, Bangkok, Thailand; 3 Department of Pharmacy Practice, College of Pharmacy, University of Illinois at Chicago, Chicago, Illinois, United States of America; 4 Department of Pharmacy Practice, Faculty of Pharmaceutical Sciences, Chulalongkorn University, Bangkok, Thailand; University of Maryland School of Medicine, UNITED STATES

## Abstract

**Background:**

Limited studies evaluate the outcome of intravenous antibiotics to oral transition in Gram-negative bloodstream infection (GN-BSI), particularly GN-BSI originating outside the urinary tract. This study aimed to evaluate treatment success in patients with GN-BSI treated with either intravenous therapy or intravenous to oral transition and to identify factors associated with treatment failure in those undergoing intravenous to oral transition.

**Methods:**

A retrospective cohort study was conducted at King Chulalongkorn Memorial Hospital, Thailand. Patients were included if they were ≥18 years of age, hospitalized in general medical wards with GN-BSI between August 1, 2015, to July 31, 2020, received intravenous antibiotic agents and had a functioning gastrointestinal tract.

**Results:**

Of 955 patients, 545 (57.1%) were in the intravenous to oral transition group. The urinary tract was the most common source of infection (38.8%). Ciprofloxacin was the most prescribed oral antibiotic (53%). Treatment success occurred in 94.3% in the intravenous antibiotic to oral transition group. There was no significant difference in treatment success between the two groups (*P* = 0.790) with a concordant result after using propensity score matching (*P* = 0.223). Independent predictors of treatment failure in the intravenous to oral transition group included metastatic solid cancer (aOR = 4.355), HIV infection with CD_4_ < 200 cells/mm^3^ (aOR = 8.452), qSOFA score ≥ 2 (aOR = 2.545), multidrug-resistant infection (aOR = 2.849), and respiratory tract infection (aOR = 8.447). Hospital length of stay in the intravenous to oral transition group was shorter than in the intravenous group (*P* < 0.001).

**Conclusions:**

Intravenous to oral transition may be a practical approach in GN-BSI. Patients with Gram-negative bacteremia who have HIV infection with CD_4_ < 200 cells/mm^3^, multidrug-resistant infections, and respiratory tract sources of infection may not be ideal candidates for this approach. Future research is needed from a randomized controlled trial.

## Introduction

Bloodstream infections (BSI) are a significant cause of morbidity and mortality in hospitalized patients [1]. The incidence of Gram-negative bloodstream infection (GN-BSI) is increasing globally, and remains a public health concern with an associated mortality of 12–25% [2–4]. Malignancy, liver cirrhosis, non-urinary tract infection, and higher Pitt bacteremia scores have been associated with higher mortality in patients with BSI [5]. Although historically GN-BSIs have been treated primarily with intravenous (IV) antibiotics, limited recent data suggest a transition from IV to oral antibiotics may be effective [6–8]. Multidrug-resistant Gram-negatives are a major public health threat around the world, especially in patients with severe infections such as BSIs. Prolonged hospitalization is associated with hospital-onset GN-BSIs and resistant pathogens [9]. Third-generation cephalosporin-resistant Enterobacterales are the main problem in southeast Asia, with an increasing trend for carbapenem-resistant Enterobacterales [10]. Data from National Antimicrobial Resistant Surveillance Center, Thailand (NARST) showed an increasing number of patients with carbapenem-resistant Enterobacterales, especially *Klebsiella pneumoniae*, during the past ten years in Thailand [11]. Antibiotic stewardship programs (ASP) can help mitigate the threat of resistant Gram negatives through systematically optimizing the use of antibiotics and have been associated with improving patient outcomes, reducing antibiotic resistance, and decreasing healthcare costs. IV to oral transition is one ASP strategy [12]. The advantages of oral antibiotics include decreasing the risk of line complications, treatment cost, and length of hospital stay (LOS) [13–16]. However, there are only limited data evaluating the clinical outcomes and factors associated with treatment failure for IV to oral transitions in GN-BSI, especially in Thai patients. This study aimed to evaluate treatment success in patients with GN-BSI treated with either IV therapy or IV to oral transition and identify predictors of treatment failure in those undergoing IV to oral transition.

## Materials and methods

### Study design, setting, and participants

A retrospective cohort study was conducted at King Chulalongkorn Memorial Hospital, a 1,479-bed tertiary referral and teaching hospital in Bangkok, Thailand. The patient inclusion criteria were as follows: (1) ≥18 years of age with GN-BSI who received inpatient care within the general medical wards from August 1, 2015, to July 31, 2020. (2) Received IV antibiotic agents. (3) Intact and functioning gastrointestinal tract, defined as the absence of the following conditions: malabsorption syndrome, short bowel syndrome, severe gastroparesis, ileus, vomiting, and continuous nasogastric suction. And (4) Available active oral antibiotics options based on susceptibilities. All patients were followed until hospital discharge. Patients were excluded if they met the following criteria: (1) Hospitalization less than 24 hours. (2) Death within 24 hours of admission. (3) Chronic infection or need for prolonged use of antibiotics, e.g., patients infected with brain abscess, septic arthritis, osteomyelitis, or infective endocarditis. (4) Uncontrolled sources of infection. (5) Patients who refused treatment or patients receiving palliative care only. (6) Patients with unavailable data in the medical record. And (7) Loss to follow up on day 30 after discharge. Patients in the IV group must have received IV antibiotics for their entire treatment duration.

The data retrieved from the paper medical records and electronic database (ePHIS) included baseline characteristics, source of infection, microorganisms, treatment, and treatment outcome. For bacterial identification, a blood sample was inoculated into an aerobic bottle of blood culture broth (BD BACTEC) and incubated at 35°C for five days. Antibiotic susceptibility testing was performed by disk diffusion and Vitek 2 XL system in accordance with the Clinical and Laboratory Standards Institute [17]. The Institutional Review Board of the Faculty of Medicine at Chulalongkorn University approved the study and waived the need for informed consent (IRB No.744/63). All data were fully anonymized before we accessed them.

### Definitions

GN-BSI was defined as a laboratory-confirmed positive blood culture with Gram-negative bacteria. The source of BSI was categorized as either primary or secondary. Secondary BSI was attributed to a body site-specific source, e.g., urinary tract, intra-abdominal tract, etc. If the BSI source could not be assigned to a specific body site despite diagnostic workup, it was classified as a primary BSI [18]. Infections identified from blood cultures taken >48 hours after admission were categorized as hospital-acquired infections, and those taken ≤48 hours after admission were categorized as community-acquired infections [19]. Sepsis and septic shock were defined according to the Third International Consensus Definition for Sepsis and Septic Shock (Sepsis 3) [20]. The Charlson comorbidity index was defined as previously described [21]. The severity of GN-BSI on day 1 of diagnosis was graded according to the Pitt bacteremia score [22–24]. Multidrug-resistant organisms were defined as resistant to at least one antibiotic from three or more classes [25]. Patients who met the following criteria on day 1 of the GN-BSI diagnosis were identified as immunocompromised: Human Immunodeficiency Virus (HIV) infection with a CD_4_ cell count of < 200 cells/mm^3^, severe neutropenia (absolute neutrophil count; ANC of < 500/mm^3^), active immunomodulatory therapy or at least 20 mg of prednisone daily or equivalent for 14 days or longer, chemotherapy within six months, or history of solid organ or hematopoietic stem cell transplant. Time to appropriate antibiotic therapy was defined as the time from identifying microorganisms to receiving antibiotics active against the microorganisms. The bioavailability of oral antibiotic agents was classified as follows: high bioavailability (>90%) included cephalexin, doxycycline, levofloxacin, moxifloxacin, and ofloxacin. Moderate bioavailability (60–90%) included ampicillin, amoxicillin, amoxicillin/clavulanic acid, ciprofloxacin, and trimethoprim/sulfamethoxazole. Low bioavailability (<60%) included ampicillin/sulbactam, cefdinir, cefditoren, cefuroxime, cefixime, and norfloxacin [26,27]. The type of IV to oral antibiotic transition was classified into three types as sequential, switch, and step-down therapy [26]. Sequential was defined as replacing an IV medication with its oral counterpart of the same compound. Switch therapy was defined as transitioning an IV medication with its oral counterpart of the same class and having the same potency level but not the same compound. Step-down therapy was defined as transitioning an IV medication with an oral agent in the spectrum of activity that may not be precisely the same. Patients were classified as having treatment success if they were afebrile (body temperature <37.8°C) with a white blood cell count (WBC) of <11,000 cells/mm^3^ after 72 hours of active antibiotic treatment and did not meet any of the treatment failure criteria. Treatment failure was a composite outcome including in-hospital mortality, relapse of BSI, and transition back from oral to IV medication. Relapse was defined as positive blood culture for the same Gram-negative microorganism within 30 days after treatment completion. Persistence of GN-BSI was defined as patients with positive blood culture for the same Gram-negative microorganism after 72 hours of active antibiotic treatment by in vitro susceptibility. Loss to follow-up on day 30 was defined as patients who didn’t return to follow up at our hospital’s clinic or receive inpatient care at our hospital on day 30 after hospital discharge.

### Statistical analysis

Means ± standard deviations (SD), medians with interquartile ranges (IQRs), and frequencies with percentages were used to describe baseline characteristics, infection source, microorganisms, type and bioavailability of oral antibiotic agents, and type of IV to oral antibiotic transitions. The Chi-square test or Fisher’s exact test was used for categorical variables. The Student t-test or the Wilcoxon rank-sum test was used for continuous data.

A logistic regression model was developed to identify factors associated with treatment failure in the overall patient population, as well as patients in the IV group and IV to oral antibiotic agents transition group. Odds ratio (ORs) and associated 95% confidence intervals (95% CI) were calculated. Variables with a *P-*value of ≤ 0.1 in the univariate analyses were included in multivariate analysis, using backward LR selection in the final model. Variables with *P*-values of < 0.05 in the multivariate analysis were considered statistically significant.

Propensity scores were calculated using a logistic regression model to balance baseline characteristics of patients in the IV group and IV to oral transition group. Covariates included in the model were preexisting medical conditions (each included separately: immunologic or rheumatologic disease, end-stage renal disease), individual immunocompromising conditions, quick Sepsis-related Organ Failure Assessment (quick SOFA) ≥ 2 on day 1 of BSI, septic shock on day 1 of BSI, ICU admission on day 1 of BSI, on mechanical ventilator on day 1 of BSI, acute kidney injury on day 1 of BSI, multidrug-resistant (MDR) pathogens, and hospital-acquired BSI. One-to-one matching without replacement and propensity variable match tolerance of 0.01 was used.

All analyses were performed using Statistical Package for the Social Science (SPSS) software for Windows, Version 22.0.

## Results

**Fig 1:**
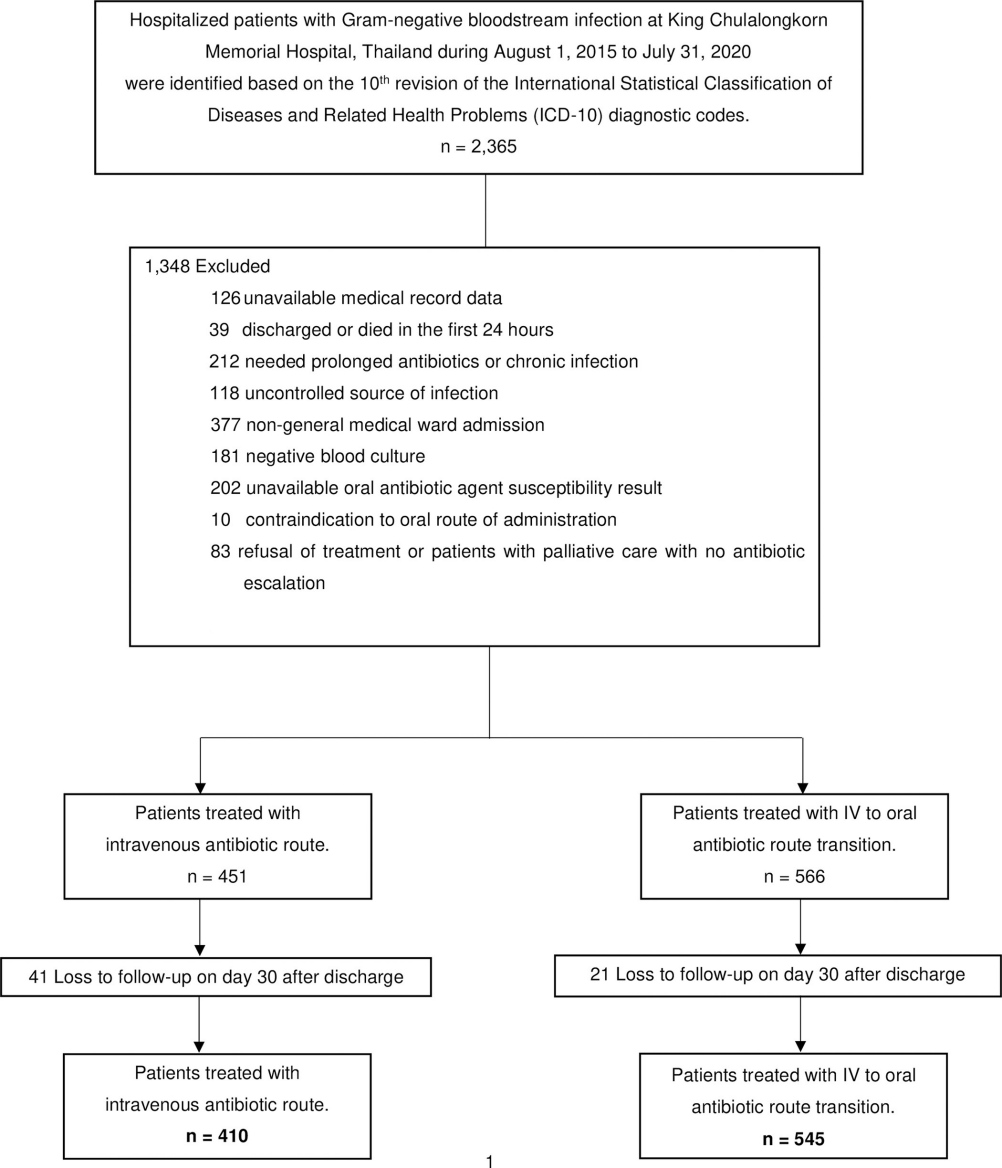
Flow diagram for patient selection.

One thousand seventeen hospitalized patients with GN-BSI met the inclusion criteria. Sixty-two patients (6.1%) were lost to follow-up on day 30 after discharge (41 patients (9.1%) were in the IV group and 21 patients (3.7%) were in the IV to oral transition group). A total of 955 patients were included in the cohort study, 410 patients (42.9%) were in the IV group, and 545 patients (57.1%) were in the IV to oral transition group (Fig 1).

Patients’ baseline characteristics are summarized in Table 1. The cohort’s median age was 71 years (IQR 60–81 years), and 547 patients (57.3%) were female. There was a higher proportion of immunocompromised hosts in the IV group than in the IV to oral transition group (32.4% versus 23.1%; *P* = 0.001). Patients in the IV group were more likely than the IV to oral transition group to be severely ill at the onset of BSI with a greater proportion having septic shock (21% versus 15.8%; *P* = 0.039), mechanical ventilation (18.5% versus 8.6%; *P* < 0.001), qSOFA scores of ≥ 2 (37.6% versus 27.3%; *P* = 0.001), and Pitt bacteremia scores of ≥ 4 (23.7% versus 9.5%; *P* < 0.001). The IV group was also more likely to have hospital-acquired infections (22.7% versus 8.8%; *P* < 0.001) and multidrug-resistant pathogens (45.4% versus 22.6%; *P* < 0.001).

**Table 1:** Baseline characteristics of hospitalized patients with Gram-negative bloodstream infection continuing intravenous antibiotic agents or receiving intravenous to oral transition antibiotics (n = 955).

Characteristics	Total (n = 955)	IV group (n = 410)	IV to PO group (n = 545)	*P-*value
Female, n (%)	547 (57.3)	249 (60.7)	298 (54.7)	0.061
Age, median (IQR) year	71 (60–81)	73 (60–82)	70 (60–81)	**0.015**
Age ≥ 65 years, n (%)	637 (66.7)	274 (66.8)	363 (66.6)	0.942
Comorbidities^a^, n (%)				
Hypertension	579 (60.6)	251 (61.2)	328 (60.2)	0.746
Diabetes mellitus	377 (39.5)	161 (39.3)	216 (39.6)	0.909
Dyslipidemia	371 (38.8)	159 (38.8)	212 (38.9)	0.970
Cardiovascular disease	258 (27.0)	118 (28.8)	140 (25.7)	0.287
Solid cancer	242 (25.3)	96 (23.4)	146 (26.8)	0.235
Metastatic solid cancer	97 (40.1)	39 (40.6)	58 (39.7)	0.889
Neurologic disease	239 (25.0)	108 (26.3)	131 (24.0)	0.416
Chronic kidney disease	162 (17.0)	71 (17.3)	91 (16.7)	0.801
Liver disease	151 (15.8)	67 (16.3)	84 (15.4)	0.697
Immunologic/Rheumatologic disease	93 (9.7)	53 (12.9)	40 (7.3)	**0.004**
Hematologic malignancy	84 (8.8)	36 (8.8)	48 (8.8)	0.988
End Stage Renal Disease	75 (7.9)	45 (11.0)	30 (5.5)	**0.002**
Chronic lung disease	62 (6.5)	23 (5.6)	39 (7.2)	0.337
HIV infection (any CD_4_)	18 (1.9)	6 (1.5)	12 (2.2)	0.406
Immunocompromised host^a^, n (%)	259 (27.1)	133 (32.4)	126 (23.1)	**0.001**
Immunomodulator or steroid within 1 month	143 (15.0)	86 (21.0)	57 (10.5)	**<0.001**
Chemotherapy within 6 months	116 (12.1)	51 (12.4)	65 (11.9)	0.810
ANC ≤ 500 cells/mm^3^	56 (5.9)	23 (5.6)	33 (6.1)	0.772
Solid organ transplant	38 (4.0)	26 (6.3)	12 (2.2)	**0.001**
HIV infection (CD_4_ < 200 cells/mm^3^)	8 (0.8)	2 (0.5)	6 (1.1)	0.478^b^
HSCT within 12 months	3 (0.3)	0 (0.0)	3 (0.6)	0.264^b^
qSOFA ≥ score 2, n (%)	303 (31.7)	154 (37.6)	149 (27.3)	**0.001**
Pitt bacteremia score ≥ 4, n (%)	149 (15.6)	97 (23.7)	52 (9.5)	**<0.001**
Charlson comorbidity index score ≥ 7, n (%)	282 (29.5)	120 (29.3)	162 (29.7)	0.878
Acute kidney injury, n (%)	402 (42.1)	199 (48.5)	203 (37.2)	**<0.001**
Lactate, median (IQR) mmol/L	2.3 (1.4–3.9)	2.3 (1.4–4.6)	2.2 (1.4–3.7)	0.537
WBC at admission, median (IQR) x10^3^ cells/mm^3^	13.8 (9.3–18.4)	13.4 (8.80–18.4)	14.1 (9.9–18.4)	0.198
Received inotropic agents, n (%)	173 (18.1)	87 (21.2)	86 (15.8)	**0.031**
Septic shock, n (%)	172 (18.0)	86 (21.0)	86 (15.8)	**0.039**
ICU admission, n (%)	98 (10.3)	72 (17.6)	26 (4.8)	**<0.001**
Length of ICU stay, median (IQR) day	4 (2.0–6.0)	4 (2.3–6.0)	3 (1.8–5.0)	0.274
Mechanical Ventilator required, n (%)	123 (12.9)	76 (18.5)	47 (8.6)	**<0.001**
Hospital acquired infection, n (%)	141 (14.8)	93 (22.7)	48 (8.8)	**<0.001**
Polymicrobial Gram-negative BSI, n (%)	45 (4.7)	25 (6.1)	20 (3.7)	0.080
Multidrug-resistant pathogens, n (%)	309 (32.4)	186 (45.4)	123 (22.6)	**<0.001**
Source of infection				
Primary BSI	211 (22.1)	101 (24.6)	110 (20.2)	0.101
Urinary tract	371 (38.8)	145 (35.4)	226 (41.5)	0.055
Intra-abdominal	248 (26.0)	90 (22.0)	158 (29.0)	**0.014**
Respiratory tract	59 (6.2)	23 (5.6)	36 (6.6)	0.527
Catheter-related	44 (4.6)	34 (8.3)	10 (1.8)	**<0.001**
Skin and soft tissue	22 (2.3)	17 (4.1)	5 (0.9)	**0.001**

**Notes:** ^a^Multiple reports possible

^b^Fisher’s exact test.

**Abbreviations:** IV, intravenous administration; PO, oral administration; IQR, interquartile range, HIV, Human Immunodeficiency Virus; ANC, absolute neutrophil count, mm^3^, cubic millimeter; HSCT, hematopoietic stem cell transplant; qSOFA, quick Sepsis-related Organ Failure Assessment; mmol/L, millimole per liters; WBC, white blood cell count; ICU, intensive care unit; BSI, bloodstream infection.

The main source of infection was urinary tract (38.8%), followed by intra-abdominal (26%) and primary BSI (22.1%). In the overall cohort, the most common microorganism was *Escherichia coli* (59.2%), followed by *Klebsiella pneumoniae* (15.8%). There was no difference in the frequency of multidrug-resistant *Escherichia coli* between the two study groups (78.5% versus 83.7%; P = 0.254). However, more patients with ceftriaxone-resistant *Escherichia coli* were present in the IV group (82.9% versus 71.8%; P = 0.043). No patients had carbapenem-resistant *Escherichia coli*. Additional microorganism data are shown in Table 2.

**Table 2:** Microorganisms of hospitalized patients with Gram-negative bloodstream infection continuing intravenous antibiotic agents or receiving intravenous to oral transition antibiotics (n = 1,004 isolates).

Microorganism (n = number of isolates)	Total (1,004 isolates)	IV group (438 isolates)	IV to PO group (566 isolates)	*P*-value
Enterobacterales, n (%)	847 (84.4)	373 (85.2)	474 (83.7)	0.541
*Escherichia coli*	594 (70.1)	254 (68.1)	340 (71.7)	0.251
*Klebsiella pneumoniae*	159 (18.8)	79 (21.2)	80 (16.9)	0.111
*Proteus mirabilis*	30 (3.5)	13 (3.5)	17 (3.6)	0.937
*Enterobact*er spp.	22 (2.6)	10 (2.7)	12 (2.5)	0.892
*Citrobacter* spp.	9 (1.1)	4 (1.1)	5 (1.1)	1.000^a^
*Klebsiella oxytoca*	9 (1.1)	0 (0)	9 (1.9)	**0.006** ^a^
*Morganella morganii*	9 (1.1)	5 (1.3)	4 (0.8)	0.518^a^
*Serratia marcescens*	5 (0.6)	4 (1.1)	1 (0.2)	0.175^a^
Others^b^	10 (1.2)	4 (1.1)	6 (1.3)	1.000^a^
Non-lactose fermenter, n (%)	92 (9.2)	46 (10.5)	46 (8.1)	0.196
*Pseudomonas aeruginosa*	45 (48.9)	19 (41.3)	26 (56.5)	0.144
*Acinetobacter baumannii*	21 (22.8)	13 (28.3)	8 (17.4)	0.214
*Acinetobacter* spp.	11 (12.0)	5 (10.9)	6 (13.0)	0.748
*Stenotrophomonas maltophilia*	7 (7.6)	3 (6.5)	4 (8.7)	1.000^a^
*Burkholderia cepacian*	5 (5.4)	5 (10.9)	0 (0)	0.056^a^
*Pseudomonas stutzeri*	3 (3.3)	1 (2.2)	2 (4.3)	1.000^a^
*Aeromonas* spp., n (%)	39 (3.9)	9 (2.1)	30 (5.3)	**0.008**
*Vibrio cholerae*, n (%)	11 (1.1)	3 (0.7)	8 (1.4)	0.365
*Hemophilus influenzae*, n (%)	6 (0.7)	1 (0.2)	5 (0.9)	0.240
Others^c^, n (%)	9 (0.9)	6 (1.4)	3 (0.5)	0.189
Multidrug-resistant pathogens; total 309 isolates, n (%)				
MDR-*Escherichia coli*	249 (80.6)	146 (78.5)	103 (83.7)	0.254
Ceftriaxone resistant *E*. *coli*	195 (78.3)	121 (82.9)	74 (71.8)	**0.043**
MDR-*Klebsiella pneumoniae*	36 (11.7)	27 (14.5)	9 (7.3)	0.054
MDR-*Enterobact*er spp.	4 (1.3)	0 (0)	4 (3.3)	**0.029** ^a^
MDR-*Acinetobacter baumannii*	4 (1.3)	3 (1.6)	1 (0.8)	1.000^a^

Notes

^a^Fisher’s exact test.

^b^Other Enterobacterales: *Edwardsiella tarda* (3), *Salmonella enterica* (3), *Providencia stuartii* (2), *Plesiomonas shigelloides* (1), *Escherichia fergusonii* (1).

^c^Other microorganisms: *Moraxella* spp. (4), *Ralstonia* spp. (2), *Rhizobium radiobacter* (1), *Shewanella putrefaciens* (1), *Sphingomonas paucimobilis* (1).

**Abbreviations:** IV, intravenous administration; PO, oral administration; MDR, multidrug-resistant; *E*. *coli*, *Escherichia coli*.

In the total population, treatment success was 94.1%, with no statistically significant between the two study groups (93.9% in the IV group versus 94.3% in the IV to oral transition group, *P* = 0.790) (Table 3). The main reason for treatment failure was infection relapse (3.5%). In-hospital mortality occurred in 0.9%. The IV to oral transition group had a significantly shorter median LOS than the IV group (7 days; IQR 5–9 days versus 15 days; IQR 10–24 days, *P* < 0.001). Six hundred fifty-seven patients (68.8%) had a follow-up blood culture after 72 hours of active antibiotic treatment. Sixteen patients (2.4%) had persistent GN-BSI. Ceftriaxone was the most common empiric therapy, followed by meropenem (47.2% and 21%, respectively).

**Table 3:** Treatment and outcomes of hospitalized patients with Gram-negative bloodstream infection treated with intravenous antibiotic agents or receiving intravenous to oral transition antibiotics (n = 955), and after propensity score matched cohort (594).

Characteristics	Full cohort (n = 955)				Propensity score matching (n = 594)		
	Total (n = 955)	IV group (n = 410)	IV to PO group (n = 545)	*P*-value	IV group (n = 297)	IV to PO group (n = 297)	*P*-value
Treatment success, n (%)	899 (94.1)	385 (93.9)	514 (94.3)	0.790	283 (95.3)	276 (92.9)	0.223
Treatment failure^a^, n (%)	56 (5.9)	25 (6.1)	31 (5.7)	0.790	14 (4.7)	21 (7.1)	0.223
Relapsed infection within 30 days	33 (3.5)	17 (4.1)	16 (2.9)		9 (3.0)	11 (3.7)	
PO to IV transition	15 (1.6)	0 (0)	15 (2.8)		0 (0)	10 (3.4)	
In-hospital mortality	9 (0.9)	8 (2.0)	1 (0.2)		5 (1.7)	1 (0.3)	
Relapsed infection within 14 days, n (%)	21 (2.2)	10 (2.4)	11 (2.0)	0.661	7 (2.4)	8 (2.7)	0.794
Duration of antibiotic therapy, median (IQR) day	14 (14–14)	14 (13–14)	14 (14–14)	0.269	14 (12–14)	14 (14–14)	**0.001**
Duration of IV antibiotic therapy, median (IQR) day	8 (5–14)	14 (13–14)	6 (4–7)	**<0.001**	14 (11–14)	6 (4–17)	**<0.001**
Length of stay, median (IQR) day	9 (6–15)	15 (10–24)	7 (5–9)	**<0.001**	14 (9–21)	7 (5–10)	**<0.001**
Inactive empirical antibiotic therapy, n (%)	148 (15.5)	84 (20.5)	64 (11.7)	**<0.001**	64 (21.5)	58 (19.5)	0.052
Time to appropriate antibiotic therapy, median (IQR) day	1 (1–1)	1 (1–1)	1 (1–1)	**<0.001**	1 (1–1)	1 (1–1)	0.400
Persistent GN-BSI, n (%)	16 (2.4)	10 (2.7)	6 (2.1)	0.646	5 (1.9)	5 (2.9)	0.470
Empirical therapy, n (%)							
Ceftriaxone	451 (47.2)	140 (34.1)	311 (57.1)	**<0.001**	125 (42.1)	136 (45.8)	0.363
Meropenem	201 (21.0)	127 (31.0)	74 (13.6)	**<0.001**	70 (23.6)	57 (19.2)	0.193
Ceftazidime	155 (16.2)	66 (16.1)	89 (16.3)	0.923	53 (17.8)	54 (18.2)	0.915
Piperacillin/tazobactam	101 (10.6)	55 (13.4)	46 (8.4)	**0.013**	37 (12.5)	36 (12.1)	0.901
Imipenem	22 (2.3)	11 (2.7)	11 (2.0)	0.498	4 (1.3)	7 (2.4)	0.361
Ciprofloxacin	14 (1.5)	4 (1.0)	10 (1.8)	0.274	3 (1.0)	4 (1.3)	1.0000^b^
Levofloxacin	6 (0.6)	5 (1.2)	1 (0.2)	0.0900^b^	2 (0.7)	1 (0.3)	1.0000^b^
Ertapenem	3 (0.3)	2 (0.5)	1 (0.2)	0.5800^b^	2 (0.7)	1 (0.3)	1.0000^b^
Amoxicillin/clavulanic acid	2 (0.2)	1 (0.2)	1 (0.2)	1.0000^b^	1 (0.3)	1 (0.3)	1.0000^b^
Combination therapy with other antibiotics, n (%)	67 (7.0)	45 (11.0)	22 (4.0)	**<0.001**	32 (10.8)	9 (3.0)	**<0.001**
Vancomycin	41 (4.3)	31 (7.6)	10 (1.8)	**<0.001**	21 (7.1)	7 (2.4)	**0.007**
Colistin	7 (0.7)	7 (1.7)	0 (0)	**0.003**0^b^	3 (1.0)	0 (0)	0.2490^b^
Fosfomycin	2 (0.2)	1 (0.2)	1 (0.2)	1.000^b^	0 (0)	0 (0)	
Antibiotic de-escalation, n (%)	336 (35.2)	161 (39.3)	175 (32.1)	**0.022**	103 (34.7)	124 (41.8)	0.076
Antibiotic escalation, n (%)	147 (15.4)	99 (24.1)	48 (8.8)	**<0.001**	79 (26.6)	36 (12.1)	**<0.001**

**Notes:** ^a^Multiple reports possible

^b^Fisher’s exact test, de-escalation: Narrower spectrum of antibiotics, escalation: Broader spectrum of antibiotics.

**Abbreviations:** IV, intravenous administration; PO, oral administration; IQR, interquartile range; GN-BSI, Gram-negative bloodstream infection.

Based on twelve covariates, one-to-one propensity score matching yielded 594 patients, with 297 patients each in the IV group and the IV to oral transition group, each with the closest propensity scores. Patients’ baseline characteristics after propensity score analysis are shown in the S1 Table. The absolute standardized difference in all baseline covariates before and after propensity score matching was ≤ 0.1 (S1 Fig).

There were notable imbalances in baseline characteristics between the two study groups before the propensity score matching. After the propensity score matching model was used, there was no statistically significant difference in treatment success in the two study groups (95.3% in the IV group versus 92.9% in the IV to oral group, *P =* 0.223). The IV to oral transition group had a shorter median hospital LOS than the IV group. A comparison of the treatment outcomes between the full cohort and propensity score-matched cohort are shown in Table 3.

**Fig 2:**
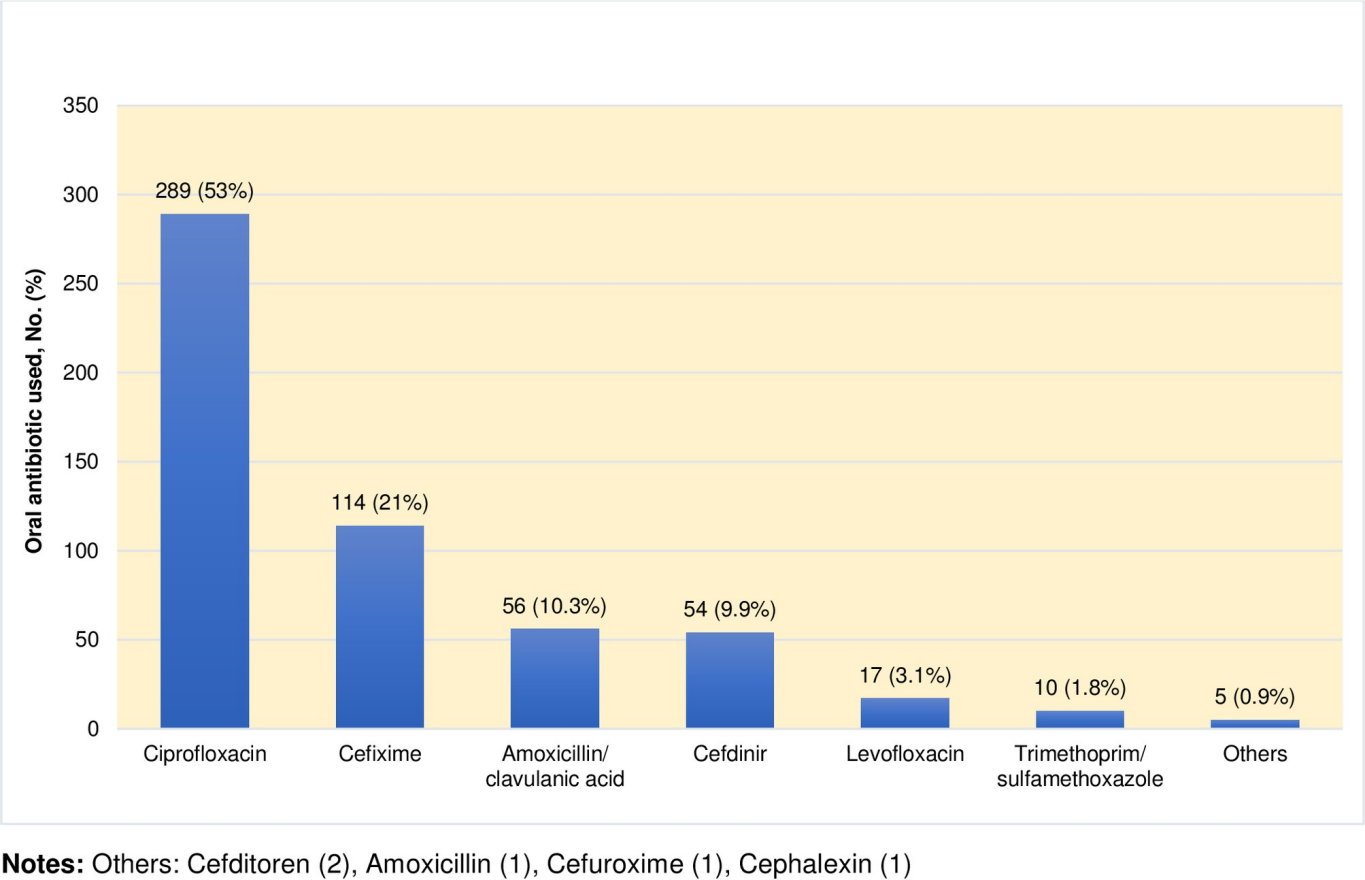
Oral antibiotic agents used in patients in the intravenous to oral antibiotic transition group (n = 545).

In the IV to oral transition group, the most common type of IV to oral transition was step-down therapy (60%), followed by switch therapy (29.5%) and sequential therapy (10.5%). Ciprofloxacin was the most frequently prescribed oral antibiotic agent (53%), followed by cefixime (21%) and amoxicillin/clavulanic acid (10.3%) (Fig 2). Most oral antibiotic agents were classified as having moderate bioavailability (356 patients; 65.3%), followed by low bioavailability (171 patients; 31.4%), and high bioavailability (18 patients; 3.3%).

The median duration of oral antibiotics was eight days (IQR 7–10 days). The median time to defervescence before IV to oral antibiotic transitions was three days (IQR 1–4 days). The median body temperature and WBC were 37.0°C (IQR 36.7–37.3°C) and 7,900 cells/mm^3^ (IQR 5,700–10,600 cells/mm^3^) on the day of transition to oral antibiotics (S2 Table).

The results of the multivariate analysis evaluating predictors of treatment failure in the IV to oral transition group are shown in Table 4; Independent predictors of treatment failure included metastatic solid cancer (aOR = 4.355, 95% CI 1.727–10.979), HIV infection with a CD_4_ count < 200 cells/mm^3^ (aOR = 8.452, 95% CI 1.150–62.114), qSOFA score of ≥ 2 (aOR = 2.545, 95% CI 1.145–5.658), multidrug-resistant pathogen (aOR = 2.849, 95% CI 1.213–6.693), and respiratory tract infection (aOR = 8.447, 95% CI 3.195–22.331). Independent predictors of treatment failure in the overall cohort included metastatic solid cancer (aOR = 3.484, 95% CI 1.649–7.361), HIV infection with CD_4_ count < 200 cells/mm^3^ (aOR = 7.359, 95% CI 1.277–42.404), qSOFA score of ≥ 2 (aOR = 2.162, 95% CI 1.217–3.843), CCI score of ≥ 7 (aOR = 2.064, 95% CI 1.056–4.034), polymicrobial GN-BSI (aOR = 2.782, 95% CI 1.091–7.095), multidrug-resistant pathogen (aOR = 2.421, 95% CI 1.362–4.305), and respiratory tract infection (aOR = 3.932, 95% CI 1.761–8.782) (S3 Table). Independent predictors of treatment failure in the IV group included metastatic solid cancer (aOR = 3.809, 95% CI 1.186–12.238), immunocompromised hosts (aOR = 3.319, 95% CI 1.262–8.734), and qSOFA score of ≥ 2 (aOR = 2.807, 95% CI 1.076–7.326) (S4 Table).

**Table 4:** Factors associated with treatment failure in hospitalized patients with Gram-negative bloodstream infection in intravenous to the oral antibiotic agent transition group (n = 545).

Factors	Univariate analysis^1^			Multivariate analysis^2^		
	OR	95% CI	*P*-value	aOR	95% CI	*P*-value
Age ≥ 65 years	1.471	0.645–3.357	0.359			
Diabetes mellitus	1.272	0.613–2.637	0.518			
Chronic kidney disease	0.519	0.154–1.744	0.289			
Cardiovascular disease	1.905	0.900–4.031	**0.092**			NS
Hematologic malignancy	1.582	0.530–4.728	0.411			
Solid cancer	2.069	0.987–4.337	**0.054**			NS
Metastatic solid cancer	3.288	1.372–7.596	**0.007**	4.355	1.727–10.979	**0.002**
HIV infection (any CD_4_)	3.476	0.728–16.601	0.118			
HIV infection (CD_4_ < 200 cells/mm^3^)	8.793	1.546–50.004	**0.014**	8.452	1.150–62.114	**0.036**
ANC ≤ 500 cells/mm^3^	1.729	0.497–6.012	0.389			
Hospital acquired infection	2.106	0.770–5.765	0.147			
qSOFA score ≥ 2	2.312	1.110–4.817	**0.025**	2.545	1.145–5.658	**0.022**
Pitt bacteremia score≥ 4	2.442	0.953–6.258	**0.063**			NS
CCI score ≥ 7	2.689	1.296–5.579	**0.008**			NS
Mechanical ventilator required	2.157	0.787–5.908	0.135			
Received inotropic agents	1.028	0.383–2.756	0.956			
Septic shock	1.028	0.383–2.756	0.956			
ICU admission	0.652	0.085–4.977	0.680			
Polymicrobial GN-BSI	3.335	0.917–12.121	**0.067**			NS
Multidrug-resistant pathogen	2.653	1.261–5.581	**0.010**	2.849	1.213–6.693	**0.016**
*Escherichia coli*	0.839	0.402–1.751	0.641			
*Klebsiella pneumoniae*	2.135	0.920–4.957	**0.077**			NS
MDR- *Klebsiella pneumoniae*	4.955	0.993–25.125	**0.051**			NS
*Pseudomonas aeruginosa*	0.652	0.085–4.977	0.680			
Intra-abdominal infection	0.572	0.230–1.421	0.229			
Urinary tract infection	0.885	0.421–1.863	0.748			
Respiratory tract infection	6.037	2.479–14.703	**<0.001**	8.447	3.195–22.331	**<0.001**
Indwelling foley catheter	1.913	0.925–3.958	**0.080**			NS
Inactive empirical antibiotic therapy	1.121	0.379–3.314	0.836			
Moderate to high bioavailability	0.615	0.294–1.285	0.196			
Low bioavailability	1.627	0.778–3.402	0.196			
Oral cephalosporins	1.642	0.785–4.343	0.187			
Oral fluoroquinolones	0.437	0.225–0.994	**0.048**			NS
Oral trimethoprim/sulfamethoxazole	1.870	0.229–15.252	0.559			
Oral amoxicillin/clavulanate	1.746	0.642–4.745	0.275			

**Notes:** ^1^Univariate analysis by Enter method

^2^Multivariate analysis by Backward LR stepwise.

**Abbreviations:** OR, Odds ratio; 95% CI, 95% confidence interval; aOR, adjusted odds ratio; NS, Non-statistically significant; HIV, Human Immunodeficiency Virus; mm^3^, cubic millimeter; ANC, absolute neutrophil count; qSOFA, quick Sepsis-related Organ Failure Assessment; CCI, Charlson comorbidity index; ICU, intensive care unit; GN-BSI, Gram-negative bloodstream infection; MDR, multidrug-resistant.

## Discussion

Our study found a high frequency of treatment success in patients with GN-BSI who received IV to oral transition. There was no significant difference in the treatment success between continuing IV antibiotic agents versus IV to oral transition in multivariate analysis in the overall cohort and our analysis with propensity score matching. This finding mirrors two previous randomized controlled trials by Amodio-Groton M et al. in bacteremic patients treated with ciprofloxacin [28] and Park TY et al. in patients who were bacteremic secondary to acute cholangitis [29]. As expected in a non-randomized study where a choice of therapy will be determined by patient clinical status, and consistent with previous findings, the group that did not undergo PO transition in our study had patients that were more immunocompromised, more severely ill, and had more hospital-acquired infections and multidrug-resistant pathogens [6,8]. Further studies are needed to support using the IV to oral transition in these groups of patients. Patients in our study’s IV to oral transition group had a shorter hospital LOS, similar to many previous studies [6–8,28]. This may have direct and indirect cost-benefit to patients and the healthcare system. Based on our results, patients with improved clinical response (afebrile and normal white blood cell count) for at least three days may be considered for IV to oral transitions. The median duration of IV antibiotic agents was six days, similar to a retrospective cohort study by Rieger KL et al. [8] and Nisly SA et al. [30]. Of note, a survey study by Thaden JT et al. [31] found most infectious disease specialists recommend extended ≥ 5 days of IV therapy before oral step-down antibiotics. The total duration of antibiotic therapy was 14 days in patients with IV to oral transition group in our study. The survey by Hospenthal DR et al. of infectious disease physician practices regarding transitioning from IV to oral therapy in patients with GN-BSI showed that 46% of responders preferred the total duration of total therapy to 14 days [32]. A systematic review and meta-analysis showed that shorter durations of antibiotic therapy in uncomplicated GN-BSI resulted in good outcomes [33]. Patients’ baseline characteristics from two randomized controlled trials were similar to our study. Most patients in the trials were less severely ill with source control, and the urinary tract was the primary source of infection [34,35]. That might explain the high treatment success in our study. Future studies are required to inform the optimal duration of treatment in patients with GN-BSI who transition from IV to oral therapy, both complicated and uncomplicated bacteremia.

The treatment failure in our study was low at 5.9%, similar to a cohort study by Rieger KL et al. [8]. However, our treatment failure was higher than a study by Thurber KM et al. [7]. They found treatment failure occurred in 2.4% in their IV group and 1.5% in their IV to oral transition group. Their study included less severe patients with GN-BSI, all of which had urinary tract sources. Our study also observed higher in-hospital mortality in the IV group, likely due to the higher severity of illness at baseline. The same trends as the study of Rieger KL et al. [8]. Our study excluded patients admitted to the non-general medical ward of 377 patients (15.9%). The non-medical general ward consisted of ICU, trauma/burn unit, orthopedic ward, and surgical ward. The main reasons we excluded the non-medical general ward were an uncontrolled source of infection followed by a need to prolong the use of antibiotics, which are essential confounders and impact our treatment outcome. We can’t generalize our result to this group of patients. The in-hospital mortality in our study was lower than in the previous study, although some patients had infections outside urinary tract sources and in the critical illness at admission [8]. This may be partly because all patients in our study had a controlled source of infection, didn’t have chronic conditions, or needed prolonged use of antibiotics.

To date, limited studies have assessed predictors of treatment failure in patients who underwent IV to oral antibiotic transition in GN-BSI. A retrospective cohort study was conducted in the USA by Kutob LF et al. to identify factors associated with treatment failure in GN-BSI patients with IV to oral transition [36]. They found four independent risk factors for treatment failure, including an immunocompromised host (adjusted hazard ratio (aHR) = 4.62), liver cirrhosis (aHR = 7.77), and patients who received a moderate (aHR = 5.94) or low bioavailability antibiotic (aHR = 7.67). Unlike previous studies, we evaluated factors associated with treatment failure in both continuing IV and IV to oral transition groups. Metastatic solid cancer and qSOFA score of ≥ 2 were associated with treatment failure in both groups; therefore, regardless of approach, these populations are more likely to have treatment failure. However, we found the IV to oral transition group had unique predictors of treatment failure that were not found in the IV group. These independent predictors of treatment failure included patients with HIV infection with CD_4_ < 200 cells/mm^3^, MDR pathogen, or respiratory tract infection. To our knowledge, no previous studies have found these three factors to be predictive of treatment failure in patients undergoing IV to oral transition. Further research is required in these patient populations. Factors associated with treatment failure in the full cohort were similar to the IV to oral transition group. In our study, more than 50% of patients were in the IV to oral transition group. The oral antibiotics used in our study differed from previous reports, given that cefixime was our second most frequently used agent [6,7,30,36,37]. Low penetration of oral third-generation cephalosporins to the epithelial lining fluid may partly explain our finding that respiratory tract infection was an independent predictor of treatment failure [38].

There was no difference in treatment failure based on the bioavailability classification of the oral agent. That is in contrast with a retrospective cohort study by Kutob LF et al., which found that treatment failure occurred in 2%, 12%, and 14% in patients receiving oral antibiotics with high, moderate, and low bioavailability, respectively (*P* = 0.02) [36]. Our study is the largest study to evaluate factors associated with treatment failure in IV to oral antibiotic transition in GN-BSI. Due to the low frequency of treatment failure in the IV to oral group, we may be underpowered to determine any impact of bioavailability on treatment failure.

Bioavailability is not the only factor associated with treatment success in GN-BSI. Antibiotic penetration at the site and pharmacodynamics, infection source, and patient’s gastrointestinal absorption will also impact treatment success. All patients in our study had an intact gastrointestinal function and received infectious source control. The main source of infection was urinary tract, which mirrors study by Nisly SA et al., comparing oral fluoroquinolones versus oral Beta-lactams versus oral trimethoprim/sulfamethoxazole in GN-BSIs and Mercuro NJ et al., comparing oral fluoroquinolones versus oral Beta-lactams in Enterobacterales BSI [30,37]. These factors also likely contributed to the lack of association of bioavailability to treatment success in our study. Moderate bioavailability agents were the most commonly prescribed in our study, similar to five previous cohort studies [6–7,30,36,37]. And ciprofloxacin was our study’s most frequently used oral agent, which is also consistent with prior studies [6–8,30,36,37]. A once-daily dose of antibiotics can improve a patient’s compliance. Levofloxacin and moxifloxacin are categorized as being highly bioavailable and also once-daily dose. Thailand is an endemic tuberculosis area, and we preserve levofloxacin and moxifloxacin for Multidrug-resistant or Extensively drug-resistant tuberculosis treatment. Ciprofloxacin was the most common oral antibiotic prescribed, followed by cefixime and amoxicillin/clavulanic acid. These three antibiotics are listed in Thailand’s national essential drug list (NELM) [39]. Thai hospitals encourage physicians to use medications in the NELM given their cost-effectiveness and universal coverage for all patients. Outpatient parenteral antimicrobial therapy (OPAT) programs can shorten LOS and improve cost-effectiveness [40]. However, OPAT is not practical for most patients at our hospital. Some patients cannot afford it or are unable to be connected with outpatient care providers to facilitate drug distribution and home nursing due to mobility issues. IV to oral transition in GN-BSI is more suitable than OPAT for our patients.

There are some strengths and limitations to our study. To our knowledge, this is the first study to evaluate the treatment success in both IV and IV to oral transition antibiotics in GN-BSI for all infection sources and microorganisms. This can reflect real-life clinical practice. Our study also identified risk factors associated with treatment failure in the IV and IV to oral transition groups. Most previous studies included only patients with Enterobacterales BSI, primarily from a urinary source. Another strength of our study, the clinical pharmacist in the medical ward advised and counseled all hospitalized patients about their discharge antibiotic therapy to improve patient adherence. We also assessed oral agents with low bioavailability, which is often not evaluated in other studies. For limitations, first, although we included a broad population of patients with GN-BSI, only a minority of patients were infected with non-lactose-fermenting Gram-negatives; thus, we cannot make general conclusions about those patients. Second, our study had fewer patients with immunocompromised status; therefore, we cannot apply our results to these specific groups of patients. Third, our study evaluated in-hospital mortality, a short-term outcome that does not capture mortality that may occur at later time points. However, our study followed-up patients for relapsed infection within 30 days after treatment completion; we might see the data for mortality during that period. Fourth, Controlling the confounding is the limitation in retrospective studies. Lastly, there were 62 patients lost to follow-up on day 30 after hospital discharge due to the inherent limitation of retrospective studies.

## Conclusion

We suggest intravenous to oral antibiotic transition be considered in patients with GN-BSIs who are hemodynamically stable with a functioning gastrointestinal tract and have source control. Careful consideration should be given to patients with HIV infection with CD4 count < 200 cells/mm3, those infected with MDR pathogens, or those with a respiratory source of infection, given these were risk factors for treatment failure. Transitioning from intravenous to oral antibiotic agents has many benefits. Still, limited data about the appropriate duration of therapy for both intravenous and oral therapy, and treatment in patient subpopulations, require additional research.

## Supporting information

S1 Fig Absolute standardized difference in all baseline covariates before and after propensity score matching. (TIF)

S1 Table Baseline characteristics of hospitalized patients with Gram-negative bloodstream infection continuing intravenous antibiotic agents or receiving intravenous to oral transition antibiotics in the full cohort (n = 955) and after propensity score matching model (n = 594). (DOCX)

S2 Table Clinical data of hospitalized patients with Gram-negative bloodstream infection in intravenous to oral antibiotic agent transitions (n = 545). (DOCX)

S3 Table Factors associated with treatment failure in hospitalized patients with Gram-negative bloodstream infection continuing intravenous antibiotic agents or receiving intravenous to oral transition antibiotics (n = 955). (DOCX)

S4 Table Factors associated with treatment failure in hospitalized patients with Gram-negative bloodstream infection continuing intravenous antibiotic agent group (n = 410). (DOCX)
