# Supplementary figures and images for: Intravenous to oral transition of antibiotics for gram-negative bloodstream infection at a University hospital in Thailand: Clinical outcomes and predictors of treatment failure
Source: PLoS One. 2022 Sep 22;17(9):e0273369. doi: 10.1371/journal.pone.0273369 (PMC9499306; doi:10.1371/journal.pone.0273369)

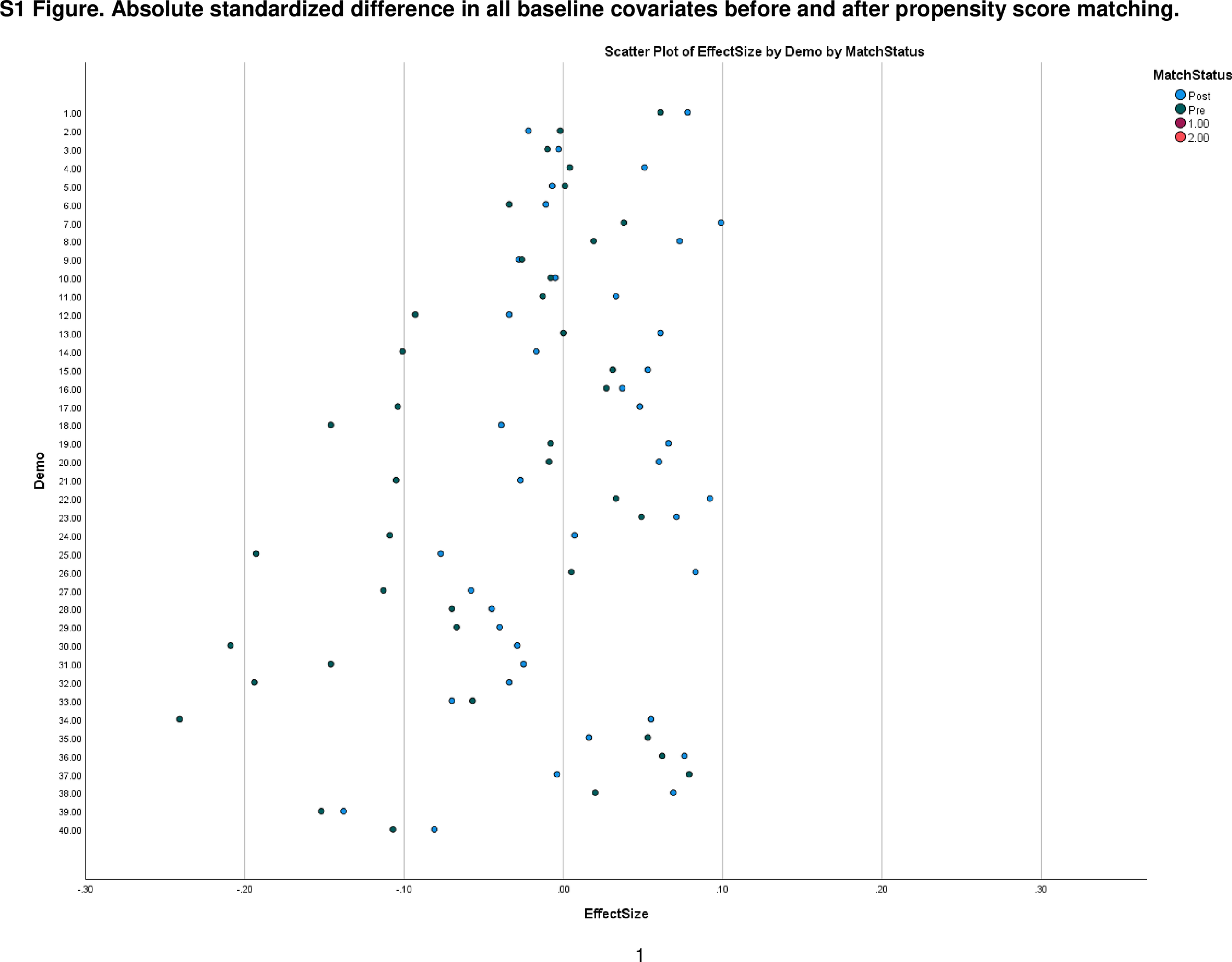

Supplement: S1 Fig — (TIF) [file pone.0273369.s001.tif]
