# Supplementary material for: Intravenous to oral transition of antibiotics for gram-negative bloodstream infection at a University hospital in Thailand: Clinical outcomes and predictors of treatment failure
Source: PLoS One. 2022 Sep 22;17(9):e0273369. doi: 10.1371/journal.pone.0273369 (PMC9499306; doi:10.1371/journal.pone.0273369)
Supplement: S1 Table — (DOCX) [file pone.0273369.s002.docx]

**S1 Table. Baseline characteristics of hospitalized patients with Gram-negative bloodstream infection continuing intravenous antibiotic agents or receiving intravenous to oral transition antibiotics in the full cohort (n=955), and after propensity score matching model (n=594).**

| **Characteristics** | **Full cohort (n=955)** | | | | | **Propensity score matching (n=594)** | | | | **No.** |
| --- | --- | --- | --- | --- | --- | --- | --- | --- | --- | --- |
|  | **Total**  **(n=955)** | **IV group**  **(n=410)** | **IV to PO group**  **(n=545)** | ***P-*value** | **Standardized effect** | **IV group**  **(n=297)** | **IV to PO group**  **(n=297)** | ***P-*value** | **Standardized effect** |  |
| Female, n (%) | 547 (57.3) | 249 (60.7) | 298 (54.7) | 0.061 | 0.061 | 182 (61.3) | 159 (53.5) | 0.056 | 0.078 | 1 |
| Age ≥ 65 years, n (%) | 637 (66.7) | 274 (66.8) | 363 (66.6) | 0.942 | -0.002 | 209 (70.4) | 203 (68.4) | 0.593 | -0.022 | 2 |
| Comorbidities^a^, n (%) | | | | | | | | | | |
| Hypertension | 579 (60.6) | 251 (61.2) | 328 (60.2) | 0.746 | -0.01 | 178 (59.9) | 177 (59.6) | 0.933 | -0.003 | 3 |
| Diabetes mellitus | 377 (39.5) | 161 (39.3) | 216 (39.6) | 0.909 | 0.004 | 114 (38.4) | 129 (43.4) | 0.211 | 0.051 | 4 |
| Dyslipidemia | 371 (38.8) | 159 (38.8) | 212 (38.9) | 0.970 | 0.001 | 115 (38.7) | 113 (38.0) | 0.866 | -0.007 | 5 |
| Cardiovascular disease | 258 (27.0) | 118 (28.8) | 140 (25.7) | 0.287 | -0.034 | 84 (28.3) | 81 (27.3) | 0.783 | -0.011 | 6 |
| Solid cancer | 242 (25.3) | 96 (23.4) | 146 (26.8) | 0.235 | 0.038 | 67 (22.6) | 93 (31.3) | **0.016** | 0.099 | **7** |
| Metastatic solid cancer | 97 (40.1) | 39 (40.6) | 58 (39.7) | 0.889 | 0.019 | 28 (9.4) | 42 (14.1) | 0.075 | 0.073 | 8 |
| Neurologic disease | 239 (25.0) | 108 (26.3) | 131 (24.0) | 0.416 | -0.026 | 75 (25.3) | 68 (22.9) | 0.502 | -0.028 | 9 |
| Chronic kidney disease | 162 (17.0) | 71 (17.3) | 91 (16.7) | 0.801 | -0.008 | 44 (14.8) | 43 (14.5) | 0.908 | -0.005 | 10 |
| Liver disease | 151 (15.8) | 67 (16.3) | 84 (15.4) | 0.697 | -0.013 | 41 (13.8) | 48 (16.2) | 0.421 | 0.033 | 11 |
| Immunologic/Rheumatologic disease | 93 (9.7) | 53 (12.9) | 40 (7.3) | **0.004** | -0.093 | 32 (10.8) | 26 (8.8) | 0.407 | -0.034 | 12 |
| Hematologic malignancy | 84 (8.8) | 36 (8.8) | 48 (8.8) | 0.988 | 0.000 | 25 (8.4) | 36 (12.1) | 0.137 | 0.061 | 13 |
| End Stage Renal Disease | 75 (7.9) | 45 (11.0) | 30 (5.5) | **0.002** | -0.101 | 31 (10.4) | 28 (9.4) | 0.681 | -0.017 | 14 |
| Chronic lung disease | 62 (6.5) | 23 (5.6) | 39 (7.2) | 0.337 | 0.031 | 17 (5.7) | 25 (8.4) | 0.200 | 0.053 | 15 |
| HIV infection (any CD_4_) | 18 (1.9) | 6 (1.5) | 12 (2.2) | 0.406 | 0.027 | 4 (1.3) | 7 (2.4) | 0.361 | 0.037 | 16 |
| Immunocompromised host^a^, n (%) | 259 (27.1) | 133 (32.4) | 126 (23.1) | **0.001** | -0.104 | 80 (26.9) | 93 (31.3) | 0.240 | 0.048 | 17 |
| Immunomodulator or steroid within 1 month | 143 (15.0) | 86 (21.0) | 57 (10.5) | **<0.001** | -0.146 | 46 (15.5) | 38 (12.8) | 0.346 | -0.039 | 18 |
| Chemotherapy within 6 months | 116 (12.1) | 51 (12.4) | 65 (11.9) | 0.810 | -0.008 | 37 (12.5) | 51 (17.2) | 0.106 | 0.066 | 19 |
| ANC ≤ 500 cells/mm^3^ | 56 (5.9) | 23 (5.6) | 33 (6.1) | 0.772 | -0.009 | 16 (5.4) | 25 (8.4) | 0.145 | 0.060 | 20 |
| Solid organ transplant | 38 (4.0) | 26 (6.3) | 12 (2.2) | **0.001** | -0.105 | 12 (4.0) | 9 (3.0) | 0.505 | -0.027 | 21 |
| HIV infection (CD_4_ < 200 cells/mm^3^) | 8 (0.8) | 2 (0.5) | 6 (1.1) | 0.478^b^ | 0.033 | 0 (0) | 5 (1.7) | 0.061^b^ | 0.092 | 22 |
| HSCT within 12 months | 3 (0.3) | 0 (0.0) | 3 (0.6) | 0.264^b^ | 0.049 | 0 (0) | 3 (1.0) | 0.249^b^ | 0.071 | 23 |
| qSOFA ≥ score 2, n (%) | 303 (31.7) | 154 (37.6) | 149 (27.3) | **0.001** | -0.109 | 93 (31.3) | 95 (32.0) | 0.860 | 0.007 | 24 |
| Pitt bacteremia score ≥ 4, n (%) | 149 (15.6) | 97 (23.7) | 52 (9.5) | **<0.001** | -0.193 | 51 (17.2) | 35 (11.8) | 0.062 | -0.077 | 25 |
| Charlson comorbidity index score ≥ 7, n (%) | 282 (29.5) | 120 (29.3) | 162 (29.7) | 0.878 | 0.005 | 84 (28.3) | 107 (36.0) | **0.043** | 0.083 | **26** |
| Acute kidney injury, n (%) | 402 (42.1) | 199 (48.5) | 203 (37.2) | **<0.001** | -0.113 | 130 (43.8) | 113 (38.0) | 0.156 | -0.058 | 27 |
| Received inotropic agents, n (%) | 173 (18.1) | 87 (21.2) | 86 (15.8) | **0.031** | -0.070 | 56 (18.9) | 46 (15.5) | 0.277 | -0.045 | 28 |
| Septic shock, n (%) | 172 (18.0) | 86 (21.0) | 86 (15.8) | **0.039** | -0.067 | 55 (18.5) | 46 (15.5) | 0.326 | -0.040 | 29 |
| ICU admission, n (%) | 98 (10.3) | 72 (17.6) | 26 (4.8) | **<0.001** | -0.209 | 30 (10.1) | 25 (8.4) | 0.479 | -0.029 | 30 |
| Mechanical Ventilator required, n (%) | 123 (12.9) | 76 (18.5) | 47 (8.6) | **<0.001** | -0.146 | 40 (13.5) | 35 (11.8) | 0.537 | -0.025 | 31 |
| Hospital acquired infection, n (%) | 141 (14.8) | 93 (22.7) | 48 (8.8) | **<0.001** | -0.194 | 45 (15.2) | 38 (12.8) | 0.407 | -0.034 | 32 |
| Polymicrobial Gram-negative BSI, n (%) | 45 (4.7) | 25 (6.1) | 20 (3.7) | 0.080 | -0.057 | 19 (6.4) | 10 (3.4) | 0.087 | -0.070 | 33 |
| Multidrug-resistant pathogens, n (%) | 309 (32.4) | 186 (45.4) | 123 (22.6) | **<0.001** | -0.241 | 107 (36.0) | 123 (41.4) | 0.178 | 0.055 | 34 |
| Source of infection | | | | | | | | | | |
| Primary BSI | 211 (22.1) | 101 (24.6) | 110 (20.2) | 0.101 | 0.053 | 232 (78.1) | 236 (79.5) | 0.688 | 0.016 | 35 |
| Urinary tract | 371 (38.8) | 145 (35.4) | 226 (41.5) | 0.055 | 0.062 | 107 (36.0) | 129 (43.4) | 0.065 | 0.076 | 36 |
| Intra-abdominal | 248 (26.0) | 90 (22.0) | 158 (29.0) | **0.014** | 0.079 | 72 (24.2) | 71 (23.9) | 0.924 | -0.004 | 37 |
| Respiratory tract | 59 (6.2) | 23 (5.6) | 36 (6.6) | 0.527 | 0.020 | 14 (4.7) | 24 (8.1) | 0.094 | 0.069 | 38 |
| Catheter-related | 44 (4.6) | 34 (8.3) | 10 (1.8) | **<0.001** | -0.152 | 29 (9.8) | 9 (3.0) | **0.001** | -0.138 | 39 |
| Skin and soft tissue | 22 (2.3) | 17 (4.1) | 5 (0.9) | **0.001** | -0.107 | 10 (3.4) | 3 (1.0) | 0.050 | -0.081 | 40 |

**Notes:** ^a^Multiple reports possible, ^b^Fisher’s exact test.

**Abbreviations:** IV, intravenous administration; PO, oral administration; IQR, interquartile range; HIV, Human Immunodeficiency Virus; ANC, absolute neutrophil count, mm^3^, cubic millimeter; HSCT, hematopoietic stem cell transplant; qSOFA, quick Sepsis-related Organ Failure Assessment; mmol/L, millimole per liters; ICU, intensive care unit; BSI, bloodstream infection.
