# Supplementary material for: Intravenous to oral transition of antibiotics for gram-negative bloodstream infection at a University hospital in Thailand: Clinical outcomes and predictors of treatment failure
Source: PLoS One. 2022 Sep 22;17(9):e0273369. doi: 10.1371/journal.pone.0273369 (PMC9499306; doi:10.1371/journal.pone.0273369)
Supplement: S2 Table — (DOCX) [file pone.0273369.s003.docx]

**S2 Table. Clinical data of hospitalized patients with Gram-negative bloodstream infection in intravenous to oral antibiotic agent transitions (n=545).**

| **Characteristics** | **IV to PO group**  **(n=545)** |
| --- | --- |
|  |  |
| Duration of oral antibiotic treatment, median (IQR) day | 8 (7 - 10) |
| Duration of afebrile until IV to Oral antibiotic agent transitions,  median (IQR) day | 3 (1 - 4) |
| Duration of normal WBC until IV to Oral antibiotic agent transitions,  median (IQR) day | 3 (1 - 5) |
| Duration with no SIRs until IV to Oral antibiotic agent transitions,  median (IQR) day | 2 (1 - 4) |
| Body temperature at IV to Oral antibiotic agent transitioning day,  median (IQR) ºC | 37.0 (36.7 - 37.3) |
| WBC at IV to Oral antibiotic agent transitioning day,  median (IQR) x10^3^cells/mm^3^ | 7.9 (5.7 – 10.6) |

**Abbreviations:** IQR, interquartile range; IV, intravenous administration; PO, oral administration; mm^3^, cubic millimeter; WBC, white blood cell count; SIRS, systemic inflammatory response syndrome.
