# Supplementary material for: Intravenous to oral transition of antibiotics for gram-negative bloodstream infection at a University hospital in Thailand: Clinical outcomes and predictors of treatment failure
Source: PLoS One. 2022 Sep 22;17(9):e0273369. doi: 10.1371/journal.pone.0273369 (PMC9499306; doi:10.1371/journal.pone.0273369)
Supplement: S3 Table — (DOCX) [file pone.0273369.s004.docx]

**S3 Table. Factors associated with treatment failure in hospitalized patients with Gram-negative bloodstream infection continuing intravenous antibiotic agents or receiving intravenous to oral transition antibiotics (n=955).**

| **Factors** | **Univariate analysis**^1^ | | | **Multivariate analysis**^2^ | | |
| --- | --- | --- | --- | --- | --- | --- |
|  | **OR** | **95% CI** | ***P*-value** | **aOR** | **95% CI** | ***P*-value** |
| IV to oral transition antibiotic | 0.929 | 0.540 – 1.599 | 0.790 |  |  |  |
| Age ≥ 65 years | 0.822 | 0.470 – 1.437 | 0.546 |  |  |  |
| Diabetes mellitus | 1.253 | 0.728 – 2.158 | 0.416 |  |  |  |
| Chronic kidney disease | 0.934 | 0.448 – 1.945 | 0.855 |  |  |  |
| Cardiovascular disease | 1.301 | 0.728 – 2.323 | 0.374 |  |  |  |
| Hematologic malignancy | 1.018 | 0.395 – 2.624 | 0.971 |  |  |  |
| Solid cancer | 3.201 | 1.854 – 5.525 | **<0.001** |  |  | NS |
| Metastatic solid cancer | 4.917 | 2.681 – 9.017 | **<0.001** | 3.484 | 1.649 – 7.361 | **0.001** |
| HIV infection (any CD_4_) | 2.044 | 0.458 – 9.119 | 0.349 |  |  |  |
| Immunocompromised host | 1.807 | 1.036 – 3.153 | **0.037** |  |  | NS |
| Chemotherapy within 6 months | 2.085 | 1.067 – 4.075 | **0.032** |  |  | NS |
| HIV infection  (CD_4_ < 200 cells/mm^3^) | 5.512 | 1.087 – 27.958 | **0.039** | 7.359 | 1.277 – 42.404 | **0.026** |
| ANC ≤ 500 cells/mm^3^ | 1.253 | 0.436 – 3.598 | 0.675 |  |  |  |
| Hospital acquired infection | 2.026 | 1.076 – 3.817 | **0.029** |  |  | NS |
| qSOFA score ≥ 2 | 2.450 | 1.423 – 4.217 | **0.025** | 2.162 | 1.217 – 3.843 | **0.009** |
| Pitt bacteremia score≥ 4 | 2.304 | 1.254 – 4.233 | **0.007** |  |  | NS |
| CCI score ≥ 7 | 3.461 | 1.999 – 5.993 | **<0.001** | 2.064 | 1.056 – 4.034 | **0.034** |
| Mechanical ventilator required | 1.510 | 0.741 – 3.077 | 0.256 |  |  |  |
| Received inotropic agents | 1.396 | 0.734 – 2.657 | 0.309 |  |  |  |
| Septic shock | 1.407 | 0.739 – 2.678 | 0.298 |  |  |  |
| ICU admission | 1.268 | 0.558 – 2.883 | 0.570 |  |  |  |
| Polymicrobial GN-BSI | 3.328 | 1.412 – 7.846 | **0.006** | 2.782 | 1.091 – 7.095 | **0.032** |
| Multidrug-resistant pathogen | 2.374 | 1.380 – 4.086 | **0.002** | 2.421 | 1.362 – 4.305 | **0.003** |
| *Escherichia coli* | 1.121 | 0.638 – 1.968 | 0.691 |  |  |  |
| *Klebsiella pneumoniae* | 1.748 | 0.930 – 3.283 | **0.083** |  |  | NS |
| MDR- *Klebsiella pneumoniae* | 2.745 | 1.024 – 7.357 | **0.045** |  |  | NS |
| *Pseudomonas aeruginosa* | 0.737 | 0.174 – 3.125 | 0.679 |  |  |  |
| Intra-abdominal infection | 1.046 | 0.568 – 1.925 | 0.886 |  |  |  |
| Urinary tract infection | 0.671 | 0.374 – 1.205 | 0.182 |  |  |  |
| Respiratory tract infection | 3.771 | 1.796 – 7.920 | **<0.001** | 3.932 | 1.761 – 8.782 | **0.001** |
| Indwelling foley catheter | 1.307 | 0.761 – 2.244 | 0.332 |  |  |  |
| Inactive empirical antibiotic therapy | 1.199 | 0.591 – 2.432 | 0.615 |  |  |  |

**Notes:** ^1^Univariate analysis by Enter method, ^2^Multivariate analysis by Backward LR stepwise

**Abbreviations:** OR, Odds ratio; 95% CI, 95% confidence interval; aOR, adjusted odds ratio; NS, Non-statistically significant; IV, intravenous administration; HIV, Human Immunodeficiency Virus; mm^3^, cubic millimeter; ANC, absolute neutrophil count; qSOFA, quick Sepsis-related Organ Failure Assessment; CCI, Charlson comorbidity index; ICU, intensive care unit; GN-BSI, Gram-negative bloodstream infection; MDR, multidrug-resistant.
