# Supplementary material for: Intravenous to oral transition of antibiotics for gram-negative bloodstream infection at a University hospital in Thailand: Clinical outcomes and predictors of treatment failure
Source: PLoS One. 2022 Sep 22;17(9):e0273369. doi: 10.1371/journal.pone.0273369 (PMC9499306; doi:10.1371/journal.pone.0273369)
Supplement: S4 Table — (DOCX) [file pone.0273369.s005.docx]

**S4 Table. Factors associated with treatment failure in hospitalized patients with Gram-negative bloodstream infection continuing intravenous antibiotic agent group (n=410).**

| **Factors** | **Univariate analysis**^1^ | | | **Multivariate analysis**^2^ | | |
| --- | --- | --- | --- | --- | --- | --- |
|  | **OR** | **95% CI** | ***P*-value** | **aOR** | **95% CI** | ***P*-value** |
| Age ≥ 65 years | 0.433 | 0.192 – 0.977 | **0.044** |  |  | NS |
| Diabetes mellitus | 1.231 | 0.544 – 2.783 | 0.618 |  |  |  |
| Liver disease | 2.106 | 0.843 – 5.262 | 0.111 |  |  |  |
| Chronic kidney disease | 1.555 | 0.598 – 4.043 | 0.366 |  |  |  |
| Cardiovascular disease | 0.770 | 0.300 – 1.978 | 0.587 |  |  |  |
| Hematologic malignancy | 0.417 | 0.055 – 3.174 | 0.398 |  |  |  |
| Solid cancer | 5.630 | 2.438 – 12.999 | **<0.001** |  |  | NS |
| Metastatic solid cancer | 8.184 | 3.377 – 19.833 | **<0.001** | 3.809 | 1.186 – 12.238 | **0.025** |
| Immunocompromise host | 2.392 | 1.060 – 5.398 | **0.036** | 3.319 | 1.262 – 8.734 | **0.015** |
| On chemotherapy within 6 months | 2.386 | 0.905 – 6.288 | **0.079** |  |  | NS |
| ANC ≤ 500 cells/mm^3^ | 0.688 | 0.089 – 5.320 | 0.720 |  |  |  |
| Hospital acquired infection | 2.016 | 0.860 – 4.724 | 0.107 |  |  |  |
| qSOFA score ≥ 2 | 2.655 | 1.161 – 6.068 | **0.021** | 2.807 | 1.076 – 7.326 | **0.035** |
| Pitt bacteremia score ≥ 4 | 2.284 | 0.991 – 5.263 | **0.053** |  |  | NS |
| CCI score ≥ 7 | 4.803 | 2.059 –11.205 | **<0.001** |  |  | NS |
| Mechanical ventilator required | 1.106 | 0.401 – 3.046 | 0.846 |  |  |  |
| Received inotropic agents | 1.823 | 0.759 – 4.377 | 0.179 |  |  |  |
| Septic shock | 1.852 | 0.771 – 4.449 | 0.168 |  |  |  |
| ICU admission | 1.526 | 0.587 – 3.968 | 0.386 |  |  |  |
| Polymicrobial Gram-negative BSI | 3.302 | 1.039 – 10.492 | **0.043** |  |  | NS |
| Multidrug-resistant pathogens | 2.248 | 0.970 – 5.214 | **0.059** |  |  | NS |
| *Escherichia coli* | 1.659 | 0.677 – 4.068 | 0.268 |  |  |  |
| MDR*- Escherichia coli* | 1.729 | 0.768 – 3.895 | 0.186 |  |  |  |
| *Klebsiella pneumoniae* | 1.373 | 0.529 – 3.560 | 0.515 |  |  |  |
| MDR- *Klebsiella pneumoniae* | 2.051 | 0.573 – 7.341 | 0.269 |  |  |  |
| *Pseudomonas aeruginosa* | 0.850 | 0.109 – 6.636 | 0.876 |  |  |  |
| Intra-abdominal infection | 2.111 | 0.900 – 4.952 | **0.086** |  |  | NS |
| Urinary tract infection | 0.438 | 0.161 - 1.191 | 0.106 |  |  |  |
| Respiratory tract infection | 1.507 | 0.333 – 6.825 | 0.594 |  |  |  |
| Indwelling foley catheter | 0.806 | 0.357 – 1.821 | 0.605 |  |  |  |
| Persistent BSI | 4.325 | 0.861 – 21.718 | **0.075** |  |  | NS |
| Inactive empirical antibiotic therapy | 1.243 | 0.480 – 3.217 | 0.654 |  |  |  |

**Notes:** ^1^Univariate analysis by Enter method, ^2^Multivariate analysis by Backward LR stepwise

**Abbreviations:** OR, Odds ratio; 95% CI, 95% confidence interval; aOR, adjusted Odds ratio; NS, Non-statistically significant; ANC, absolute neutrophil count; mm^3^, cubic millimeter; qSOFA, quick Sepsis-related Organ Failure Assessment; CCI, Charlson comorbidity index; ICU, intensive care unit; BSI, bloodstream infection; MDR, multidrug-resistant.
